# Could ^18^F-FES PET Be a New Driver in Therapeutic Choice for Metastatic HR+/HER2− Patients?

**DOI:** 10.3390/diagnostics15172139

**Published:** 2025-08-25

**Authors:** Maria Vita Sanò, Alessandro Russo, Lorenza Marino, Sarah Pafumi, Martina Di Pietro, Giuseppina Rosaria Rita Ricciardi

**Affiliations:** 1 Department of Medical Oncology, Humanitas Istituto Clinico Catanese, 95045 Misterbianco, Italy; mariavita.sano@humanitascatania.it (M.V.S.); alessandro.russo@hunimed.eu (A.R.); lorenza.marino@humanitascatania.it (L.M.); sarah.pafumi@humanitascatania.it (S.P.); 2 Department of Biomedical Sciences, Humanitas University, 20027 Pieve Emanuele, Italy; 3 Department of Onco-Haematology, Papardo Hospital, 98158 Messina, Italy; martinadipietro@aopapardo.it

**Keywords:** ^18^F-FES PET, breast cancer, Luminal, ET, endocrine therapy, FDG-PET, CDK 4/6, HR+/HER2−

## Abstract

Hormone receptor-positive (HR+)/human epidermal growth factor receptor 2-negative (HER2−) breast cancer is the most prevalent subtype. Positron emission tomography (PET) imaging with 16α-18F-fluoro-17β-fluoroestradiol (^18^F-FES), a radiolabeled form of estradiol, enables the assessment in vivo of ER expression, ER heterogeneity in metastatic sites and functionally active ER capable of ligand binding. This imaging modality has been recently approved as a diagnostic tool for detecting ER-positive lesions in patients with recurrent or metastatic breast cancer. Despite promising activity, the role of this powerful tool is still debated. Herein we critically analyzed current evidence supporting the use of 18F-FES PET in metastatic ER+/HER2− breast cancer, highlighting the potential challenges for clinical implementation.

## 1. Introduction

Hormone receptor-positive (HR+)/human epidermal growth factor receptor 2-negative (HER2−) breast cancer is the most prevalent subtype, comprising approximately 70% of new cases. Initial treatment of ER+/HER2− metastatic breast cancer (mBC) includes endocrine therapy (ET) as the preferred option [1,2]. Recently, the therapeutic landscape of HR+/HER2− has dramatically changed with the approval of novel effective therapies that demonstrated improved outcomes in this setting [3,4]. 

Positron emission tomography (PET) imaging with 16α-18F-fluoro-17β-fluoroestradiol (^18^F-FES), a radiolabeled form of estradiol, enables the assessment in vivo of ER expression, ER heterogeneity in metastatic sites and functionally active ER capable of ligand binding. This imaging modality can inform therapeutic decision-making, potentially minimizing the use of ineffective endocrine therapies in HR+/HER2− mBC. On May 2020, the U.S. Food and Drug Administration (FDA) approved ^18^F-FES PET/CT as a diagnostic tool for detecting ER-positive lesions in patients with recurrent or metastatic breast cancer. 

## 2. ^18^F-FES PET in HR+/HER2− mBC

**Figure 1:**
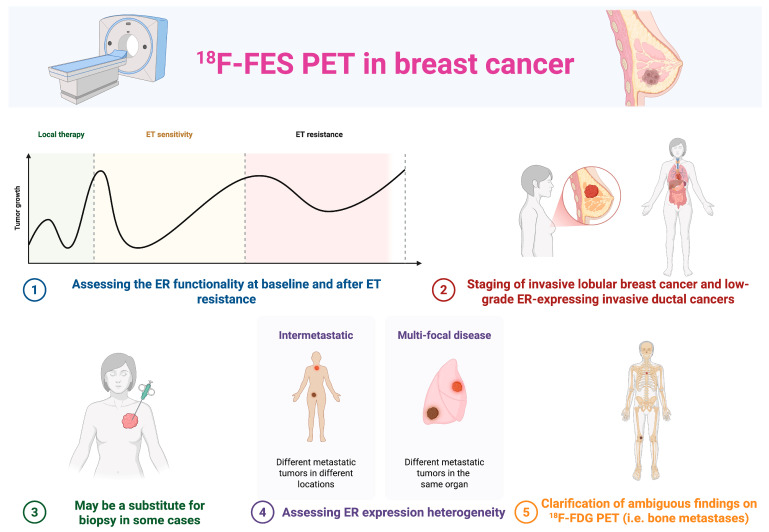
Potential application of the ^18^F-FES PET in HR+/HER2− breast cancer.

The Society of Nuclear Medicine and Molecular Imaging (SNMMI) convened an expert workgroup to evaluate the literature and establish appropriate use criteria (AUC). The workgroup concluded that the most appropriate uses of ^18^F-FES PET include assessing ER functionality when considering endocrine therapy, either at the initial diagnosis of metastatic breast cancer or after disease progression on endocrine therapy. ^18^F-FES PET/CT may be helpful in the staging of invasive lobular breast cancer (which is often less visible with conventional imaging techniques) and low-grade ER-expressing invasive ductal cancers and may be a substitute for biopsy in some cases [5,6]. HR+/HER2− mBC patients typically receive cyclin-dependent kinase 4/6 inhibitors (CDK4/6i) combined with endocrine therapy as first-line treatment. While this approach yields prolonged progression-free survival (PFS) in most patients, approximately 20% experiences rapid progression despite CDK4/6i. ER expression heterogeneity across metastatic sites contributes significantly to endocrine resistance, highlighting the need for whole-body ER assessment (Figure 1) [7,8]. 

## 3. Clinical Development of ^18^F-FES PET/CT

The phase 2 ET-FES trial assessed ^18^F-FES PET/CT in distinguishing endocrine-sensitive from endocrine-resistant mBC based on SUV thresholds. ER+/HER2− mBC patients with FES-SUV >2 were classified as endocrine-sensitive and received endocrine therapy (ET), while those with FES-SUV <2 were randomized to ET (Arm A) or chemotherapy (Arm B). At 62.4 months follow-up, mPFS was 18 months for SUV >2, 12.4 months in Arm A, and 23 months in Arm B. mOS was not reached for SUV >2, while it was 28.2 months in Arm A and 52.8 months in Arm B. It is worth noting that the study enrolled ‘true’ endocrine-sensitive patients (first-line treatment, long disease-free interval, de novo mBC) [9]. The trial did not meet the primary objective, which was to compare the activity of first-line ET versus first-line ChT in mBC patients with ER+/HER2− mBC and ^18^F-FES SUV < 2 at baseline CT/PET scan:

- Primary endpoint was the disease control rate (DCR), as defined by the proportion of non-progressing patients within 3 months of treatment:
- Secondary objectives were to assess the DCR with ET in patients with ^18^F-FES SUV ≥ 2; the DCR with ET in patients with ^18^F-FES SUV ≥ 2 vs. those with ^18^F-FES SUV < 2; the ER expression in the primary tumor and overall ^18^F-FES-uptake in metastases; and, finally, the OS in all patients and by ^18^F-FES SUV value.

The trial was prematurely closed due to the SARS-CoV-2 pandemic, and the production and delivery of 18F-FES from the manufacturing site of production was stopped on December 2020, after an overall enrollment of 147 patients.

The results of the ET-FES trial are limited in the endocrine-resistant group by the inclusion as ET of single-agent hormonotherapy without the addition of CD4/6 inhibitors, which might have been beneficial for selected patients with endocrine resistance on an ^18^F-FES SUV <2. An important finding of the ET-FES trial is the different response to single-agent AI as compared to selective estrogen receptor modulators (SERMs) such as tamoxifen or selective estrogen receptor downregulators (SERDs) like fulvestrant, as the use of AI in patients with endocrine-sensitive disease, with a mean ^18^F-FES SUV ≥2, was significantly higher than fulvestrant or tamoxifen [9]. 

A retrospective study analyzed the intrapatient heterogeneity of ^18^F-FES PET as a prognostic factor in 102 patients with mBC and demonstrated that patients with ER-positive homogenous expression showed a trend toward longer outcomes as compared with those with heterogenous expression [10,11]. 

Following CDK4/6 inhibitors, second-line treatment options for ER+/HER2− mBC now include next-generation selective estrogen receptor degraders, PI3K/AKT inhibitors, CDK inhibitors, and antibody–drug conjugates (ADCs), such as trastuzumab deruxtecan (T-DXd). Notably, treatment with T-DXd demonstrated superior PFS compared to the physician’s choice of chemotherapy in patients with rapid progression (<6 months) and primary endocrine resistance on first-line ET plus CDK4/6 inhibitors. The ongoing ESTROTIMP trial in France is evaluating the impact of ^18^F-FES PET/CT on therapeutic decisions post-CDK4/6 inhibitor therapy. Interim analysis of 30 enrolled patients, presented at the San Antonio Breast Cancer Symposium (SABCS) 2024, indicated that, in 11 cases, treatment plans were altered based on FES PET/CT results [12]. These findings support the incorporation of ^18^F-FES PET/CT in the baseline diagnostic work-up of ER+/HER2− mBC to allow the identification of a subset of patients classified as endocrine-resistant based on a mean SUV, where the upfront administration of first-line tailored therapy can improve outcomes. It can help to assess HR status in lesions that are difficult to biopsy but mainly could inform post-CDK4/6i therapeutic decisions, where there is a strong unmet need for predictive biomarkers.

## 4. Limitations and Future Perspectives

Studies have shown that ^18^F-FES PET clarifies ambiguous findings on ^18^F-FDG PET/CT, including bone metastases evaluation, with potential impact on patient management [13]. However, a potential limitation of the ^18^F-FES PET/CT is the high liver uptake, due to its physiologic excretion through the hepatobiliary system [6], albeit the use background correction and separate thresholds can allow ER status determination with ^18^F-FDG PET/CT in most liver metastases [14]. In addition, false positive cases should be excluded as non-breast cancer conditions, such as uterine leiomyoma, meningioma, atelectasis, pneumonitis, and interstitial lung disease, can be associated with ^18^F-FDG PET/CT uptake. Beyond these biological aspects, other major limitations for ^18^F-FDG PET/CT clinical practice introduction might be the costs and availability of this approach and logistic issues. Moreover, the analysis of ^18^F-FES PET/CT should be homogeneously shared among the different nuclear medicine facilities and feasible, as for ^18^F-FDG PET. Furthermore, medication interference can be an issue, as long washout periods are required for certain drugs (8 weeks for tamoxifen and 28 weeks for fulvestrant), which can be impractical in some clinical situations [15]. In conclusions, currently available evidence suggests that ^18^F-FES PET/CT might be a valuable tool in breast cancer management in selected cases, for ER expression assessment and treatment decision-making. However, several technical and biological limitations do not provide supportive evidence for a widespread use of this methodology. The results of ongoing studies evaluating ^18^F-FES PET in multiple clinical settings will provide definitive conclusions on this promising molecular imaging and the right place in the diagnostic algorithm of ER+/HER2− breast cancer. 

## Data Availability

Not applicable.

## Abbreviations

The following abbreviations are used in this manuscript:

mBC	Metastatic breast cancer
HR+	Hormone receptor-positive
HER2−	Human epidermal growth factor receptor 2-negative
PET	Positron emission tomography
18F-FES	16α-18F-fluoro-17β-fluoroestradiol
FDA	Food and Drug Administration
CDK4/6i	Cyclin-dependent kinase 4/6 inhibitors
CT	Computed tomography
ET	Endocrine therapy
SUV	Standardized uptake value

## References

[1] Jhaveri K., Marmé F. (2024). Current and Emerging Treatment Approaches for Hormone Receptor-Positive/Human Epidermal Growth Factor Receptor 2-Negative Metastatic Breast Cancer. Cancer Treat. Rev..

[2] Cazzaniga M.E., Pronzato P., Amoroso D., Bernardo A., Biganzoli L., Bisagni G., Blasi L., Bria E., Cognetti F., Crinò L. (2023). Clinical Outcomes of HER2−Negative Metastatic Breast Cancer Patients in Italy in the Last Decade: Results of the GIM 13-AMBRA Study. Cancers.

[3] Sanò M.V., Martorana F., Lavenia G., Rossello R., Prestifilippo A., Sava S., Ricciardi G.R., Vigneri P. (2022). Ribociclib Efficacy in Special Populations and Analysis of Patient-Reported Outcomes in the MONALEESA Trials. Expert. Rev. Anticancer. Ther..

[4] Lloyd M.R., Jhaveri K., Kalinsky K., Bardia A., Wander S.A. (2024). Precision Therapeutics and Emerging Strategies for HR-Positive Metastatic Breast Cancer. Nat. Rev. Clin. Oncol..

[5] Ulaner G.A., Mankoff D.A., Clark A.S., Fowler A.M., Linden H.M., Peterson L.M., Dehdashti F., Kurland B.F., Mortimer J., Mouabbi J. (2023). Summary: Appropriate Use Criteria for Estrogen Receptor-Targeted PET Imaging with 16α-(18)F-Fluoro-17β-Fluoroestradiol. J. Nucl. Med..

[6] Mankoff D., Balogová S., Dunnwald L., Dehdashti F., DeVries E., Evangelista L., Van Kruchten M., Vaz S.C., Fowler A., Linden H. (2024). Summary: SNMMI Procedure Standard/EANM Practice Guideline for Estrogen Receptor Imaging of Patients with Breast Cancer Using 16α-[(18)F]Fluoro-17β-Estradiol PET. J. Nucl. Med..

[7] Goetz M.P., Toi M., Campone M., Sohn J., Paluch-Shimon S., Huober J., Park I.H., Trédan O., Chen S.-C., Manso L. (2017). MONARCH 3: Abemaciclib As Initial Therapy for Advanced Breast Cancer. J. Clin. Oncol..

[8] Hortobagyi G.N., Stemmer S.M., Burris H.A., Yap Y.-S., Sonke G.S., Paluch-Shimon S., Campone M., Blackwell K.L., André F., Winer E.P. (2016). Ribociclib as First-Line Therapy for HR-Positive, Advanced Breast Cancer. N. Engl. J. Med..

[9] Gennari A., Brain E., De Censi A., Nanni O., Wuerstlein R., Frassoldati A., Cortes J., Rossi V., Palleschi M., Alberini J.L. (2024). Early Prediction of Endocrine Responsiveness in ER+/HER2−Negative Metastatic Breast Cancer (MBC): Pilot Study with (18)F-Fluoroestradiol ((18)F-FES) CT/PET. Ann. Oncol..

[10] van Geel J.J.L., Moustaquim J., Boers J., Elias S.G., Smeets E.M.M., Knip J.J., Glaudemans A.W.J.M., de Vries E.F.J., Hospers G.A.P., van Kruchten M. (2025). Intrapatient 16α-[(18)F]Fluoro-17β-Estradiol PET Heterogeneity as a Prognostic Factor for Endocrine Therapy Response and Survival in Patients with Estrogen Receptor-Positive Metastatic Breast Cancer. J. Nucl. Med..

[11] Jeong H., Ryu J., Jeong J.H., Han S., Hyung J., Ahn J.-H., Jung K.H., Kim S.-B., Jeong B.-K., Lee H.J. (2025). Predictive and Prognostic Value of (18)F-FES PET/CT for Patients with Recurrent or Metastatic Breast Cancer Treated with Endocrine Therapy plus Cyclin-Dependent Kinase 4/6 Inhibitors. Eur. J. Nucl. Med. Mol. Imaging.

[12] Bidard F.-C., Bidard, Seban R.-D., van de Ven S., Ladoire S., Bellesoeur A., Parisse-Di Martino S., Humbert O., Cassou-Mounat T., Deshayes E. (2025). Abstract PS11-02: [18F]Fluoroestradiol (FES) PET/CT to Guide 2nd Line Treatment Decision in Patients with ER-Positive HER2−Negative Advanced Breast Cancer (ABC) Progressing on 1st Line Aromatase Inhibitor and CDK4/6 Inhibitor: Early Results of the ESTROTIMP Trial. Clin. Cancer Res..

[13] Guglielmo P., Mazzola R., Darwish S.S., Valenti F., De Pas T.M., Setti L., Bonacina M., Grassi M.M., Evangelista L. (2025). Head-to-Head Comparison of [(18)F]FES and [(18)F]FDG PET/CT in Breast Cancer Patients: Has a New Era Come?. Eur. J. Nucl. Med. Mol. Imaging.

[14] Boers J., Loudini N., de Haas R.J., Willemsen A.T.M., van der Vegt B., de Vries E.G.E., Hospers G.A.P., Schröder C.P., Glaudemans A.W.J.M., de Vries E.F.J. (2021). Analyzing the Estrogen Receptor Status of Liver Metastases with [(18)F]-FES-PET in Patients with Breast Cancer. Diagnostics.

[15] Dey R., Cepeda De Jesus G., Karak P. (2024). Navigating Uncertainty with 18-F FES PET/CT: Modern Solutions to Diagnostic Dilemmas in Breast Cancer Patients. J. Nucl. Med..

